# Anemia, Glycemia, and Inflammation in PAD-Related Tissue Necrosis

**DOI:** 10.3390/metabo16090662

**Published:** 2026-09-09

**Authors:** Mehmet Aslan

**Affiliations:** Department of Cardiovascular Surgery, Alanya Education and Research Hospital, 07400 Antalya, Turkey; mehmetaslan81@hotmail.com; Tel.: +90-537-023-4424

**Keywords:** peripheral artery disease, tissue necrosis, anemia, admission hyperglycemia, red blood cell-to-albumin ratio, limb salvage

## Abstract

**Objective:** To determine whether systemic inflammation in peripheral artery disease is primarily associated with necrosis or a secondary response, we evaluated the association of tissue oxygenation (red blood cells), acute metabolic stress (admission glucose, albumin), systemic inflammation (C-reactive protein), and the red blood cell-to-albumin ratio. **Materials and Methods:** We retrospectively analyzed 375 patients undergoing endovascular interventions (58 with necrosis, 317 without). C-reactive protein-to-albumin and red blood cell-to-albumin ratios were calculated. Associations were assessed using parallel multivariate logistic regression models. **Results:** The necrosis group had significantly higher admission glucose and C-reactive protein-to-albumin ratio, but a lower red blood cell count and red blood cell-to-albumin ratio. In multivariate models of fundamental biomarkers, C-reactive protein lost independent significance; low red blood cells (Odds Ratio: 0.348, *p* = 0.0003) and elevated admission glucose (Odds Ratio: 1.008, *p* = 0.0018) were the strongest independently associated variables. In composite index models, the C-reactive protein-to-albumin ratio became a dominant associated factor, attenuating the red blood cell-to-albumin ratio’s statistical significance. **Conclusions:** Anemia and admission hyperglycemia are strong correlates of tissue necrosis. Systemic inflammation appears to act as a secondary consequence of ischemic injury but profoundly exacerbates tissue loss when combined with nutritional depletion. Comprehensive medical management, including anemia correction and metabolic optimization, is fundamental for limb salvage.

## 1. Introduction

Peripheral artery disease (PAD) is a systemic and progressive manifestation of atherosclerosis, representing a major cause of cardiovascular morbidity characterized by chronic limb-threatening ischemia (CLTI) and irreversible tissue necrosis in its advanced stages [1,2]. Current American Heart Association/American College of Cardiology (AHA/ACC) and European Society of Cardiology (ESC) guidelines emphasize the importance of systemic risk factor modification and the optimization of biochemical parameters, in addition to anatomical revascularization, in limb salvage strategies [2,3,4].

The relationship between PAD prognosis and inflammatory and nutritional markers, such as C-reactive protein (CRP) and albumin, has been frequently reported in the literature [5,6,7]. However, it remains debated whether the inflammatory response observed in ischemic wounds acts as a primary driver of vascular pathology or a secondary immune response to tissue ischemia. While macrovascular arterial occlusion initiates chronic limb-threatening ischemia (CLTI), the ultimate progression to irreversible tissue necrosis is heavily dictated by microcirculatory failure and systemic physiological reserves. Specifically, reduced oxygen-carrying capacity—often manifesting clinically as anemia—and acute metabolic stress, such as admission hyperglycemia, profoundly exacerbate cellular hypoxia in the compromised capillary beds. However, the exact interplay between these systemic vulnerabilities and the localized inflammatory response requires further elucidation.

In this study, the association of fundamental parameters representing tissue oxygenation potential (red blood cell [RBC] count), acute metabolic stress (admission glucose), and repair capacity (albumin) with the development of tissue necrosis was investigated. For this purpose, the independent correlations and diagnostic accuracy of the RBC-to-Albumin Ratio (RCAR), which reflects tissue repair reserve, and the CRP-to-Albumin Ratio (CAR), which reflects systemic inflammatory burden, in distinguishing the presence of necrosis were analyzed.

## 2. Materials and Methods

This retrospective cohort study includes 375 patients selected from a large cohort who underwent endovascular interventions with a diagnosis of peripheral artery disease in our clinic between 2022 and 2025. The study protocol was approved by the Local Ethics Committee of the affiliated university and was conducted in accordance with the principles of the Declaration of Helsinki.

**Figure 1:**
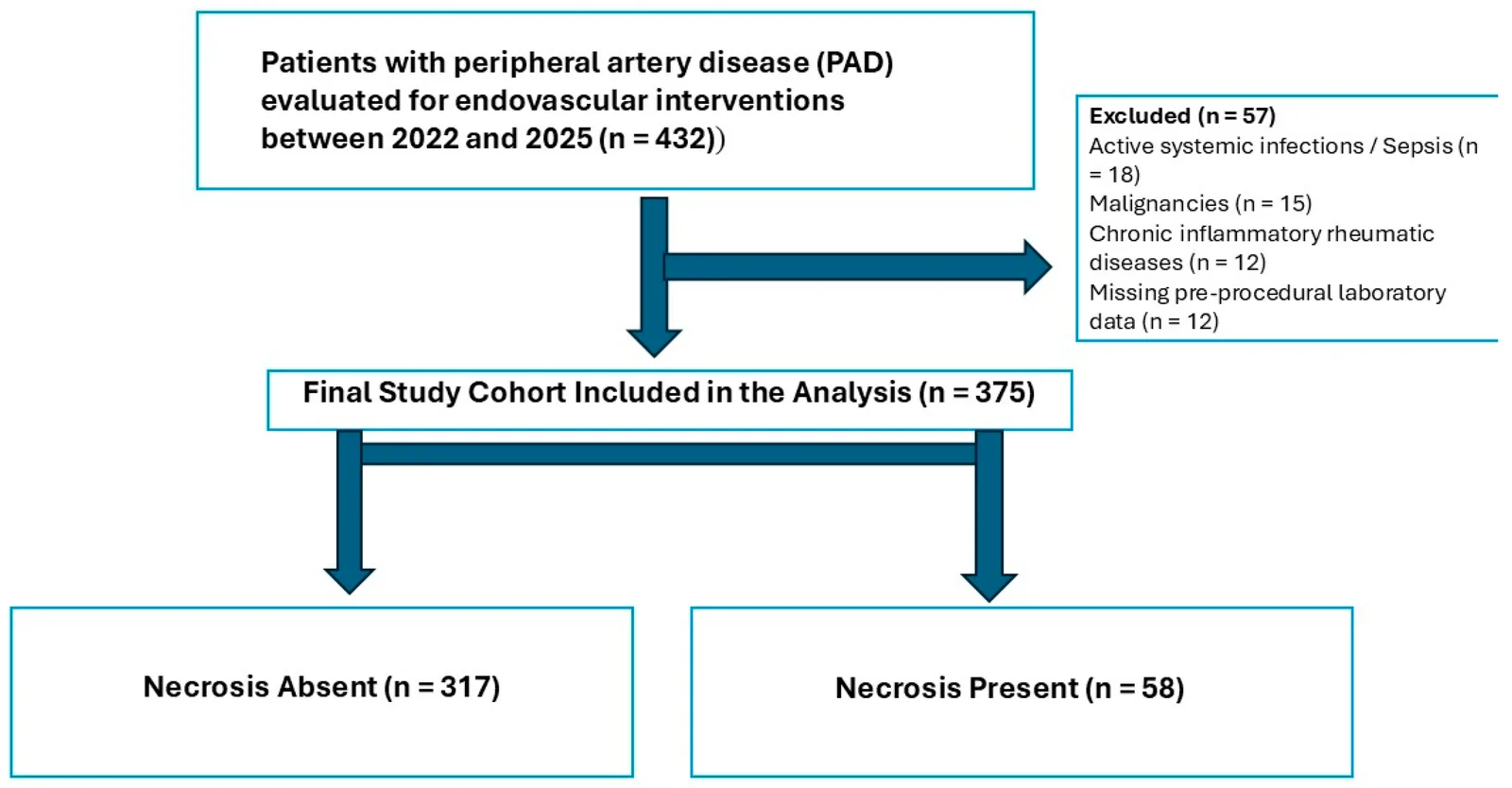
STROBE flow diagram of the study cohort selection process. The flowchart details the initial number of patients evaluated, specific exclusion criteria (including active systemic infections, malignancies, and missing data), and the final categorization of the 375 included patients based on the presence of tissue necrosis.

Patients were divided into two groups based on the presence of tissue necrosis/loss (according to clinical examination findings at the time of admission and the Rutherford classification): Necrosis Present (n = 58) and Necrosis Absent (n = 317) (Figure 1). Due to the retrospective nature of the study, complete historical data to calculate contemporary WIfI (Wound, Ischemia, foot Infection) scores were not uniformly available; therefore, local wounds were assessed clinically. Biomarkers were obtained from the routine pre-procedural laboratory data of all patients upon initial hospital admission. Serum CRP (mg/L), Albumin (g/L), admission glucose (mg/dL), and RBC count (10^6^/µL) values were recorded. Standard C-reactive protein (CRP) was utilized instead of high-sensitivity CRP (hs-CRP) as it aligns with the routine acute-admission biomarker protocol in our institution. To evaluate the systemic inflammatory and metabolic status, CAR and RCAR were calculated. Individuals with active systemic infections (defined by clinical signs of sepsis, profound leukocytosis, or positive blood cultures), malignancies, or chronic inflammatory rheumatic diseases were excluded from the study.

### Statistical Analysis

The conformity of the data to a normal distribution was evaluated using both the Kolmogorov–Smirnov and Shapiro–Wilk tests. Due to the non-normal distribution of the data confirmed by both tests, the Mann–Whitney U test was used for the comparison of continuous variables between the groups. Categorical variables were analyzed using the Chi-square test. To determine the independent association of the parameters with the presence of necrosis and to prevent multicollinearity between fundamental biomarkers and their derived indices, parallel multivariate logistic regression models (Enter method) were established. The diagnostic accuracy and discriminatory power of the biomarkers were calculated using Receiver Operating Characteristic (ROC) curve analysis and Area Under the Curve (AUC) values. The limit of statistical significance was accepted as *p* < 0.05. All analyses were performed using IBM SPSS Statistics v26.0 (IBM Corp., Armonk, NY, USA) and GraphPad Prism v8.4 software (GraphPad Software, San Diego, CA, USA).

## 3. Results

The mean age of the 375 patients included in the study was found to be 65.37 ± 10.68 years. When the patients were stratified according to the presence of necrosis, no statistically significant difference was observed in terms of age between the necrosis absent (n = 317) and necrosis present (n = 58) groups (65.27 vs. 65.91 years, *p* = 0.840) (Table 1). When baseline clinical characteristics and comorbidities were evaluated, the overall prevalence in the study cohort was 68.2% for smoking history, 17.3% for diabetes mellitus, 15.0% for hyperlipidemia, and 5.3% for chronic kidney disease (CKD). Importantly, there were no statistically significant differences between the necrosis absent and necrosis present groups regarding the distribution of these baseline variables (*p* > 0.05 for all), ensuring that the groups were clinically well-balanced.

### 3.1. Analysis of Laboratory Parameters

**Figure 2:**
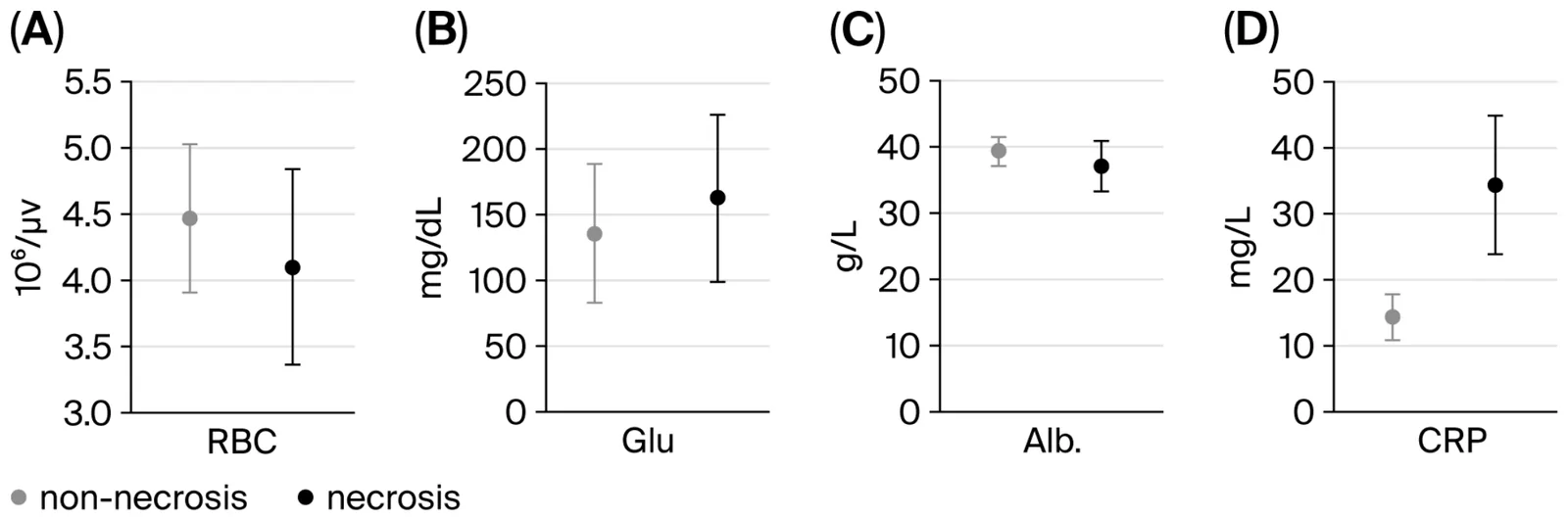
Comparison of fundamental biomarker levels according to the presence of necrosis. The graphs illustrate the mean and error margin (standard deviation/95% confidence interval) values of the key laboratory parameters between the patient groups without necrosis (necrosis absent) and with necrosis (necrosis present). Panels represent the distribution of (**A**) RBC count (× 10^6^/µL), (**B**) Admission Glucose (mg/dL), (**C**) Albumin (g/L), and (**D**) CRP (mg/L) levels.

Admission glucose (166.7 ± 51.3 vs. 136.1 ± 51.0 mg/dL, *p* = 0.001) and CRP (47.9 ± 32.9 vs. 23.7 ± 24.0 mg/L, *p* < 0.001) levels at admission were found to be significantly higher in the necrosis group. In contrast, RBC values were significantly lower in the necrosis group compared to the control group (4.00 ± 0.69 vs. 4.45 ± 0.58 × 10^6^/µL, *p* < 0.001). When albumin levels were examined, the serum albumin levels of the patients in the necrosis group were observed to be significantly lower (37.13 ± 3.82 vs. 39.28 ± 2.78 g/L, *p* < 0.001) (Figure 2).

**Figure 3:**
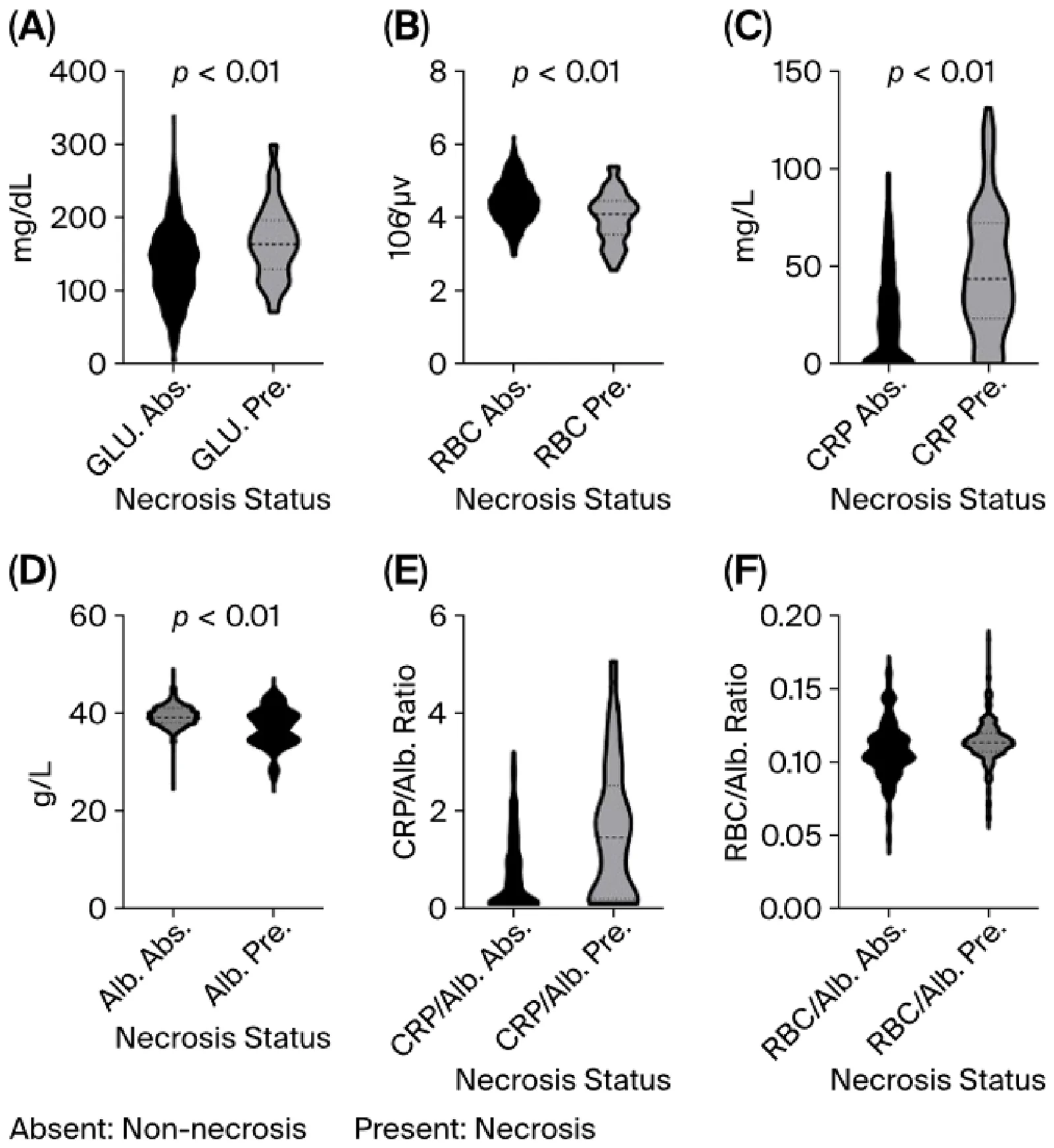
Distribution of the investigated biomarkers in the study group according to necrosis status. The distributions of the parameters (**A**) Admission Glucose, (**B**) RBC, (**C**) CRP, (**D**) Albumin, (**E**) CRP-to-Albumin Ratio (CAR), and (**F**) RBC-to-Albumin Ratio (RCAR) between the necrosis absent (n = 317) and necrosis present (n = 58) groups are presented using violin plots. The plots represent the median, interquartile range, and distribution density. *p*-values indicating significant differences in intergroup comparisons are provided on the graphs.

Upon analyzing the calculated ratios, the CRP-to-Albumin Ratio (CAR) was found to be significantly higher in the necrosis group (1.61 ± 1.38 vs. 0.74 ± 0.76, *p* < 0.001), whereas the RBC-to-Albumin Ratio (RCAR) was significantly lower in the necrosis group (0.108 ± 0.022 vs. 0.114 ± 0.016, *p* < 0.001). No significant differences were detected between the groups in terms of urea, creatinine, sodium, and calcium levels (Table 1, Figure 3). Although the mean creatinine level was mathematically higher in the necrosis group (1.79 ± 2.10 mg/dL), it was accompanied by a remarkably large standard deviation. This wide variance is mathematically attributable to a statistical right-skewness caused by the small subset of patients (5.3%) with advanced CKD, rather than a generalized deterioration in renal function across the entire necrosis group, thus explaining the lack of statistical significance (*p* = 0.557).

### 3.2. Diagnostic Accuracy and ROC Analysis

**Figure 4:**
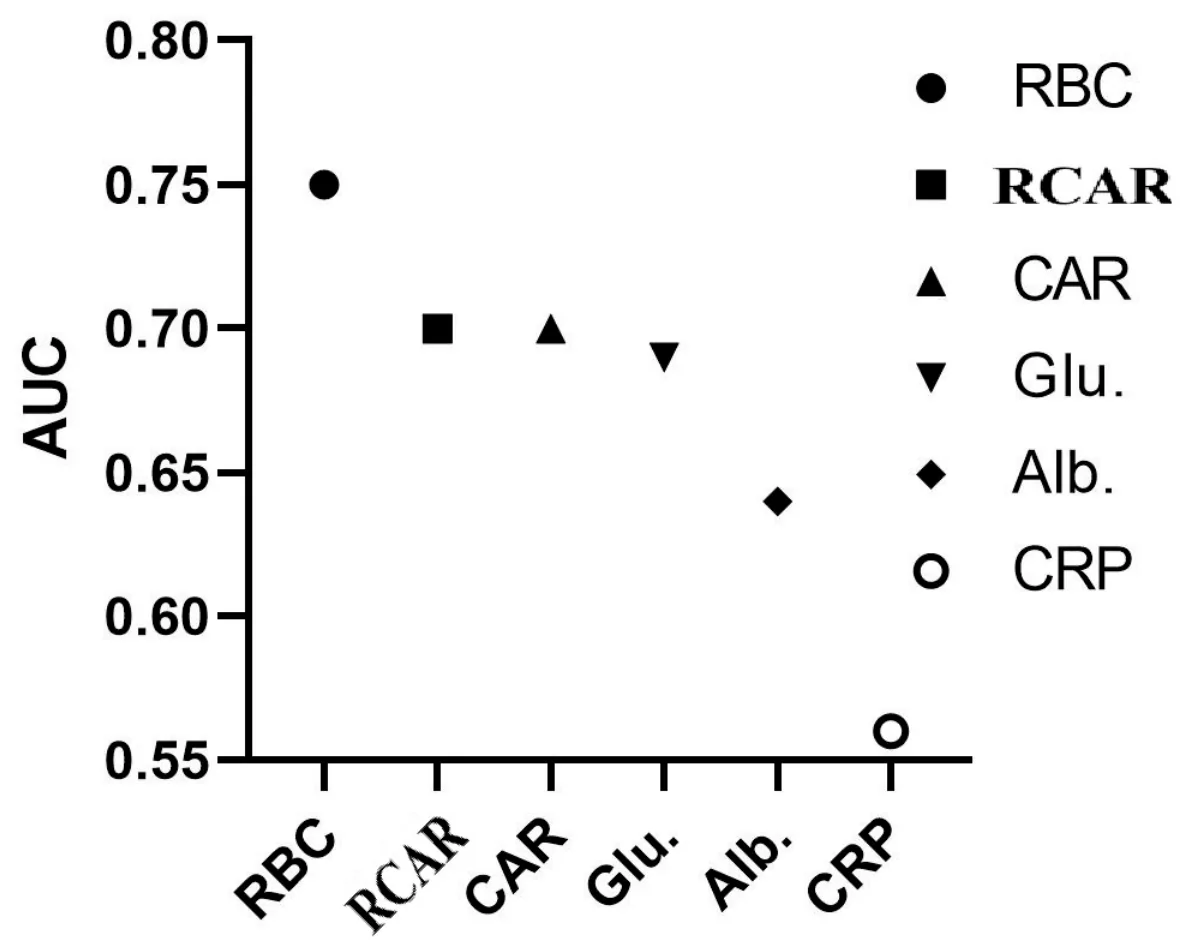
Comparison of Area Under the Curve (AUC) values for the investigated biomarkers. This graph comparatively presents the AUC values obtained from the ROC analysis used to predict the development of tissue necrosis: RBC: With an AUC value of 0.75, it is the parameter with the highest diagnostic accuracy in the graph. Being the dot closest to the upper left corner highlights its strong predictive value for the presence of necrosis. CRP-to-Albumin Ratio (CAR) and RBC-to-Albumin Ratio (RCAR): Both ratios exhibit very close and strong predictive performances, each with an AUC value of 0.70. Admission Glucose: With an AUC value of 0.69, it demonstrates clinically significant accuracy in predicting necrosis. CRP: With an AUC value of 0.56, it is the parameter closest to the diagonal reference line in the graph. This indicates that the power of CRP alone in discriminating necrosis is weak.

**Figure 5:**
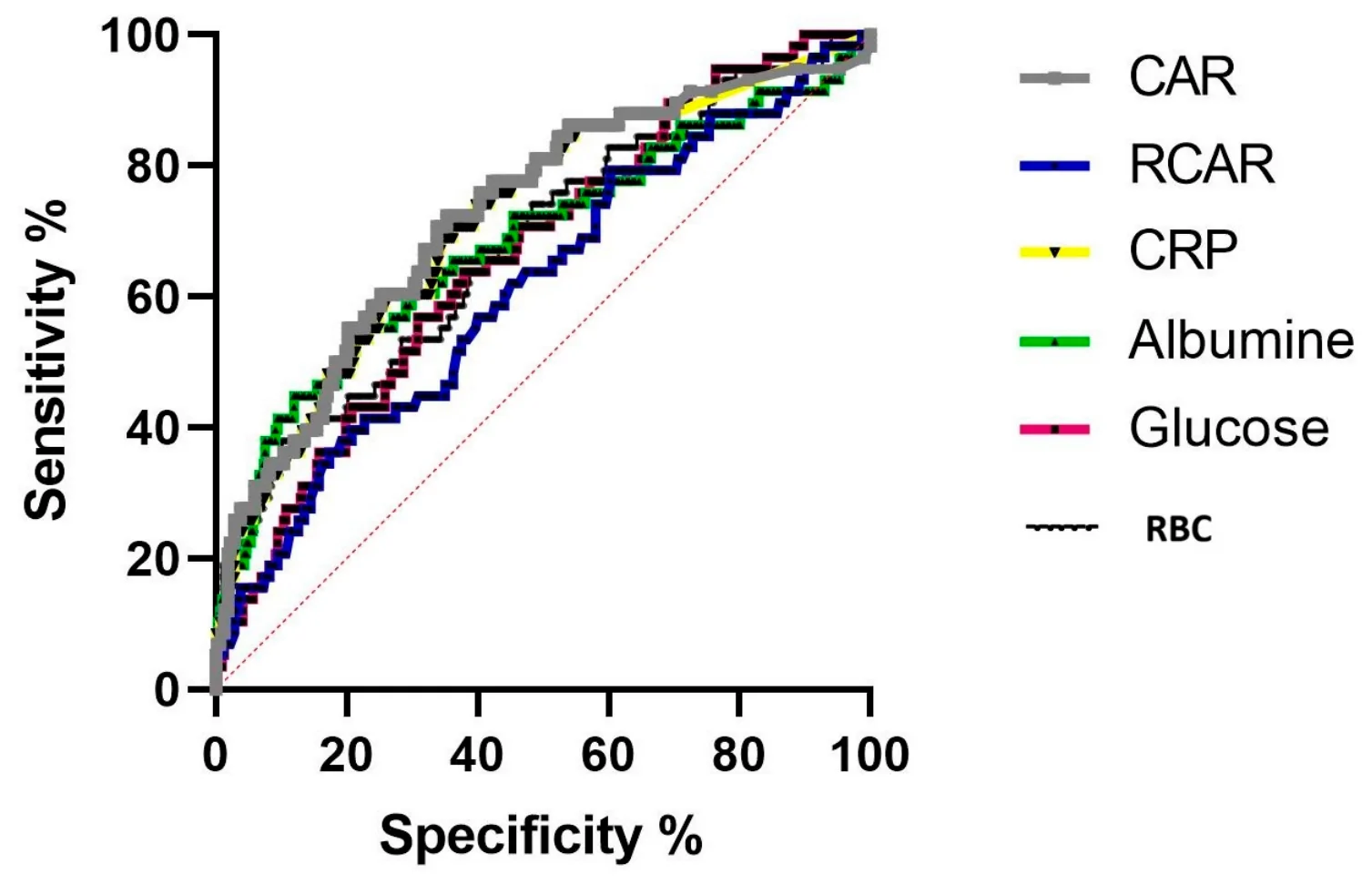
Receiver Operating Characteristic (ROC) curves of the evaluated biomarkers. The graph illustrates the diagnostic performance (sensitivity vs. 100–specificity) of the six fundamental parameters (RBC, Admission Glucose, Albumin, CRP, CAR, and RCAR) in predicting the development of tissue necrosis.

The diagnostic performances of the biomarkers in predicting the presence of necrosis were evaluated using ROC analysis (Figure 3). The RBC parameter was determined to have the highest discriminatory power (AUC: 0.75, 95% CI: 0.68–0.82, *p* < 0.001). Both RCAR (AUC: 0.70) and CAR (AUC: 0.70) exhibited similar and moderate but clinically meaningful values for estimating necrosis. While the diagnostic accuracy of admission glucose was determined as AUC: 0.69 (demonstrating modest accuracy), the success of the CRP parameter alone in discriminating necrosis was observed to be limited (AUC: 0.56, *p* = 0.185) (Table 2). The ROC analysis presented in Figure 3 reveals the weak power of the systemic inflammatory response (CRP) in predicting necrosis (AUC: 0.56), contrasted by the diagnostic superiority of RBC (0.75) and Albumin-based ratios (0.70); thereby strongly highlighting the critical role of anemia and metabolic reserve in the necrosis process (Figure 4). The detailed diagnostic performance metrics, including the sensitivity and specificity values for these six fundamental parameters, are presented in Figure 5.

### 3.3. Logistic Regression and Correlation Analysis

**Figure 6:**
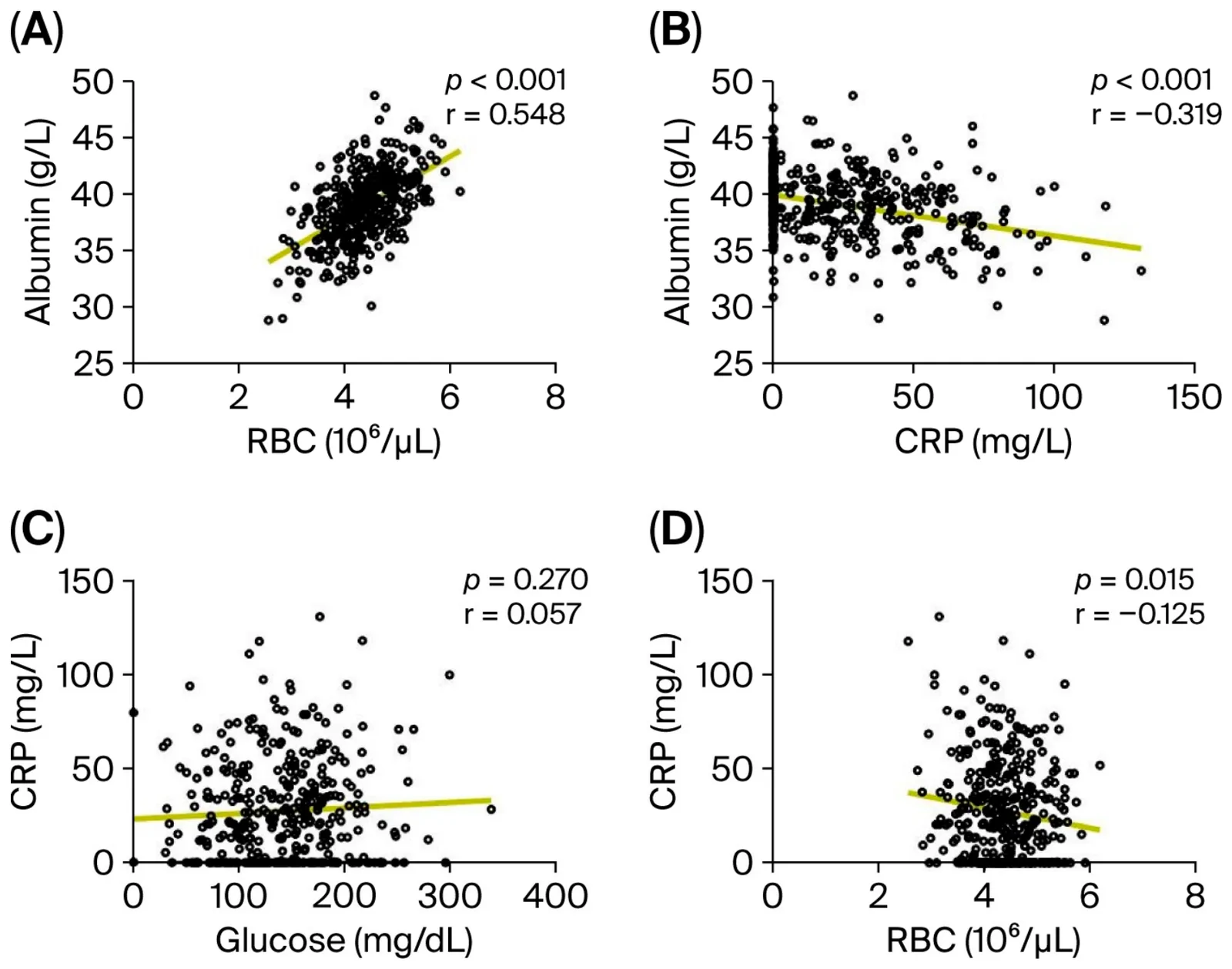
Correlation analysis of the relationships between fundamental parameters. The linear relationships between the main biomarkers investigated in the study are analyzed using scatter plots and regression lines: (**A**) RBC and Albumin: Positive correlation between tissue repair reserve and oxygenation capacity (*r* = 0.548, *p* < 0.001). (**B**) CRP and Albumin: Inverse relationship between inflammatory burden and biochemical reserve (*r* = −0.319, *p* < 0.001). (**C**) Admission Glucose and CRP: The impact of acute metabolic stress on the systemic inflammatory response (*r* = 0.057, *p* = 0.270). (**D**) RBC and CRP: Correlation between tissue ischemia/anemia and the inflammatory response, suggestive of a secondary response (*r* = −0.125, *p* = 0.015). The Pearson correlation coefficient (r) and *p*-values in the graphs represent the strength and significance of the relationships between the variables.

To prevent multicollinearity between fundamental biomarkers and their mathematical combinations, multivariate logistic regression analysis was conducted using two parallel models (Table 3). In Model 1, which evaluated fundamental parameters, isolated RBC (OR: 0.348, 95% CI: 0.196–0.617, p = 0.0003) and admission glucose (OR: 1.008, 95% CI: 1.003–1.013, p = 0.0018) were identified as factors independently associated with necrosis, while age and CRP did not show an independent effect. In Model 2, which evaluated composite indices, the CRP-to-Albumin Ratio (CAR) emerged as a strongly associated factor (OR: 3.650, 95% CI: 2.392–5.568, *p* < 0.001), alongside admission glucose (OR: 1.011, 95% CI: 1.004–1.018, *p* = 0.002). When adjusted for CAR and admission glucose in Model 2, the independent predictive power of RCAR did not reach statistical significance (*p* = 0.137). Furthermore, correlation analysis between fundamental biomarkers revealed a significant inverse relationship between RBC and CRP (*r* = −0.125, *p* = 0.015), and a positive correlation between RBC and Albumin (*r* = 0.548, *p* < 0.001), mathematically supporting the hypothesis that systemic inflammation likely develops as a secondary response to tissue ischemia and anemia (Figure 6).

## 4. Discussion

Tissue necrosis and CLTI are the most important harbingers of limb loss in individuals with PAD [1,4,8]. The most striking finding of our study is the observation that anemia and admission hyperglycemia, rather than isolated systemic inflammation, emerged as independent fundamental associated factors for the development of necrosis. In our multivariate analysis (Model 1), it was determined that every 1-unit decrease in the RBC value increased the likelihood of necrosis approximately 3-fold (OR: 0.348, *p* = 0.0003). It appears that anemia potentially accelerates the transition from the ischemia phase to the necrosis phase by impairing tissue oxygenation. This situation confirms the critical role of Patient Blood Management (PBM) strategies, which aim to manage anemia and preserve biological reserve in patients with ischemic wounds, during limb salvage processes [9,10].

Another strong factor independently associated with necrosis development is elevated admission glucose (OR: 1.008, *p* = 0.0018). The relationship between glycemic management and PAD outcomes is highly complex. The ACCORD trial demonstrated that an intensive glucose control strategy reduced amputations and microvascular complications, although overly aggressive targets raised concerns in specific cohorts [11]. In contrast, the ADVANCE trial showed a reduced risk of microvascular complications without an increase in overall death rates with intensive glucose control [12]. In the modern era, as emphasized by recent ACC statements on diabetic management in patients with PAD [2], the focus has shifted towards the ‘cardiometabolic continuum’. Novel pharmacological agents, such as Glucagon-like peptide-1 receptor agonists (GLP-1ra) and Sodium-glucose cotransporter-2 inhibitors (SGLT2i), exert synergistic, pleiotropic effects [4]. Beyond merely lowering glucose, these agents directly mitigate systemic inflammation, improve endothelial function, and provide profound cardiovascular and limb protection. While elevated admission glucose impairs the microvascular bed and hinders wound healing, we acknowledge that it may not solely reflect chronic poor glycemic control. Although HbA1c is the traditional gold standard for chronic diabetes management, its numerically restricted scale and delayed kinetics may not fully capture the acute metabolic crisis in patients presenting with CLTI. In this context, admission glucose acts as a highly sensitive and dynamic barometer, reflecting both the underlying chronic dysregulation and the acute “stress hyperglycemia” triggered by severe ischemic pain, infection, and tissue damage.

Although it is frequently stated in the literature that inflammatory indices such as CAR [7], Systemic Immune-Inflammation Index (SII) [13,14], and Neutrophil-to-Lymphocyte Ratio (NLR) [15,16] correlate with prognosis in patients with PAD, our parallel multivariate analysis reveals a more complex pathophysiological dynamic. While isolated systemic inflammation (CRP) failed to show independent association with necrosis in Model 1 (*p* = 0.534), supporting its role as a secondary response, it transformed into a profound exacerbating factor of tissue loss when coupled with a diminished biochemical repair reserve (low albumin). Specifically, the CRP-to-Albumin Ratio (CAR) emerged as a dominant independently associated factor in Model 2 (OR: 3.650, *p* < 0.001). Furthermore, while RCAR serves as a highly sensitive diagnostic marker demonstrating the inadequacy of tissue oxygenation and repair reserve (0.108 ± 0.022 vs. 0.114 ± 0.016, *p* < 0.001), it lost its independent statistical significance when adjusted for CAR and admission glucose. This mathematical phenomenon suggests that while isolated anemia (RBC) is fundamentally associated with ischemic necrosis, the ultimate collapse of the tissue is predominantly linked to the synergistic destructive effect of systemic inflammation and catabolic depletion (CAR). This thesis is also supported by the clinical findings of Ferreira et al., who observed that the high systemic inflammatory profile detected in chronic limb-threatening ischemia (CLTI) cases regressed and improved following successful revascularization [5].

The counterpart of these findings in clinical practice is clear: anatomical revascularization alone (whether bypass or endovascular, supra- or infra-popliteal) is not sufficient to prevent limb loss in patients with PAD. Optimal limb salvage mandates comprehensive medical therapy—including aggressive lipid-lowering, modern antidiabetic agents, and supervised exercise therapy—in conjunction with mechanical intervention. While the BEST-CLI trial demonstrated the impact of the revascularization strategy on clinical success in patients with CLTI [17], the BASIL-2 trial highlighted the roles of different strategies on amputation-free survival in patients requiring infra-popliteal revascularization [18]. However, regardless of the revascularization strategy, as emphasized by the WIfI (Wound, Ischemia, foot Infection) classification endorsed by the Society for Vascular Surgery [19] and the staging systems in the Global Vascular Guidelines (GVG) [8], a successful and durable limb salvage does not seem possible without achieving systemic optimization of the patient (treatment of anemia, mitigation of inflammatory-nutritional depletion, and aggressive metabolic control).

It is crucial to acknowledge that the cross-sectional design of our study limits the ability to draw definitive causal inferences. Because tissue necrosis and the analyzed biomarkers were assessed simultaneously at hospital admission, we cannot definitively establish whether altered RBC counts or elevated inflammatory indices preceded the onset of necrosis or developed as a consequence of it. Therefore, while anemia and metabolic stress are strongly associated with advanced ischemia, prospective longitudinal studies are required to confirm their temporal roles in tissue loss.

## 5. Conclusions

The strongest fundamental correlates of tissue necrosis in patients with PAD are anemia and admission hyperglycemia. Systemic inflammation alone is not the primary independent factor associated with necrosis; however, when combined with nutritional and biochemical depletion (as measured by CAR), it becomes a profound exacerbating factor of tissue loss. A low RCAR serves as a valuable auxiliary clinical indicator, demonstrating the inadequacy of tissue repair capacity. The success of limb salvage strategies must rely not only on the mechanical opening of the vessel but also on comprehensive medical therapy, supervised exercise, aggressive treatment of anemia, nutritional optimization, and rigorous metabolic management starting from the preoperative period.

## 6. Limitations

Our study has several important limitations. First, the retrospective, single-center design restricts our ability to establish definitive causality and may limit generalizability. Second, the current multivariate analysis could not comprehensively adjust for several major clinical and anatomical determinants, such as detailed infra-popliteal lesion distribution, exact WIfI staging, and complete medication histories (e.g., specific usage of statins, SGLT2i, or GLP-1ra). Therefore, the observed associations may partly reflect a more advanced CLTI phenotype rather than exclusively independent biological effects. Third, glycemic status was determined using admission glucose rather than longitudinal HbA1c data. Although HbA1c is the gold standard for assessing chronic glycemic control, its measurement reliability is profoundly compromised in patients with severe anemia and altered red blood cell turnover—which emerged as a core pathophysiological characteristic in our ischemic cohort. Furthermore, since elevated admission glucose also captures “acute stress hyperglycemia” secondary to severe ischemic pain, infection, or peri-admission stress, our ability to strictly differentiate chronic glycemic dysregulation from acute metabolic stress is limited. Therefore, pathophysiological conclusions regarding admission glucose and necrosis development should be interpreted cautiously. Fourth, regarding the evaluation of tissue oxygenation, our multivariable models primarily utilized the absolute RBC count. While the strict clinical diagnosis of anemia relies on hemoglobin or hematocrit thresholds, verification analysis within our dataset confirmed a highly significant correlation between absolute RBC counts and hemoglobin levels (r = 0.89, *p* < 0.001). This indicates that in our specific cohort, the reduced RBC count robustly and directly paralleled the clinical anemic state. Therefore, the term ‘anemia’ used throughout this study encompasses this clinically verified reduction in circulating erythrocyte density and oxygen-carrying capacity. Finally, biomarker levels were assessed only at admission, precluding the evaluation of dynamic physiological changes following revascularization.

## Figures and Tables

**Table 1:** Comparison of Demographic and Admission Laboratory Characteristics of the Study Cohort According to the Presence of Necrosis: Data are expressed as mean ± standard deviation or counts (percentages). The Mann–Whitney U test was used for continuous variables and the Chi-square test for categorical variables. (*) Indicates a statistically significant difference (*p* < 0.05). RBC: Red Blood Cell count, CRP: C-Reactive Protein.

Parameter	Necrosis Absent (n = 317)	Necrosis Present (n = 58)	*p*-Value
Demographics & Clinical Characteristics			
Age (years)	65.27 ± 10.89	65.91 ± 9.42	0.840
Smoking, n (%)	216 (68.1%)	40 (69.0%)	0.903
Diabetes Mellitus, n (%)	55 (17.4%)	10 (17.2%)	0.985
Hyperlipidemia, n (%)	48 (15.1%)	8 (13.8%)	0.794
Chronic Kidney Disease, n (%)	17 (5.4%)	3 (5.2%)	0.956
Laboratory Findings			
Admission Glucose (mg/dL)	136.1 ± 51.0	166.7 ± 51.3	<0.001 *
RBC (×10^6^/µL)	4.45 ± 0.58	4.00 ± 0.69	<0.001 *
Albumin (g/L)	39.28 ± 2.78	37.13 ± 3.82	<0.001 *
C-Reactive Protein (mg/L)	23.7 ± 24.0	47.9 ± 32.9	<0.001 *
Urea (mg/dL)	41.07 ± 14.47	53.72 ± 36.92	0.330
Creatinine (mg/dL)	1.02 ± 0.46	1.79 ± 2.10	0.557
Sodium (mEq/L)	138.24 ± 2.50	137.04 ± 4.19	0.063
Calcium (mg/dL)	9.21 ± 0.53	9.18 ± 0.68	0.746
Calculated Ratios			
RBC-to-Albumin Ratio (RCAR)	0.114 ± 0.016	0.108 ± 0.022	<0.001 *
CRP-to-Albumin Ratio (CAR)	0.74 ± 0.76	1.61 ± 1.38	<0.001 *

**Table 2:** Diagnostic Performance of Biomarkers in Discriminating the Presence of Necrosis (ROC Analysis Results): Receiver Operating Characteristic (ROC) curve analysis results are presented. AUC: Area Under the Curve, CI: Confidence Interval. An AUC value significantly greater than 0.5 indicates diagnostic power. The RBC parameter exhibited the highest diagnostic accuracy, followed closely by the RCAR and CAR parameters, in predicting the presence of necrosis.

Biomarker	AUC	95% Confidence Interval	*p*-Value
RBC	0.75	0.681–0.819	<0.001
RBC-to-Albumin Ratio (RCAR)	0.70	0.622–0.778	<0.001
CRP-to-Albumin Ratio (CAR)	0.70	0.625–0.775	<0.001
Admission Glucose	0.69	0.605–0.775	0.005
Albumin	0.64	0.556–0.724	<0.001
CRP	0.56	0.468–0.652	0.185

**Table 3:** Multivariate Logistic Regression Analysis of Independent Risk Factors: * To prevent multicollinearity, parallel models were established. While RBC and Admission Glucose were independent predictors in Model 1, the composite inflammatory-nutritional index (CAR) emerged as a dominant independent risk factor in Model 2, overpowering the independent predictive value of RCAR.

Parameter	Odds Ratio (OR)	95% Confidence Interval	*p*-Value
Fundamental Biomarkers			
RBC count	0.348	0.196–0.617	0.0003 *
Admission Glucose	1.008	1.003–1.013	0.0018 *
CRP	1.002	0.994–1.011	0.534
Age	0.999	0.967–1.033	0.988
Composite Indices *			
RCAR (per 0.01 unit)	0.816	0.624–1.067	0.137
CAR	3.650	2.392–5.568	<0.001 *
Admission Glucose	1.011	1.004–1.018	0.002 *

## Data Availability

The data presented in this study are available on request from the corresponding author. The data are not publicly available due to privacy and ethical restrictions regarding patient confidentiality.
